# Perioperative Microbiologic Monitoring of Sputum on Postoperative Day One as a Predictor of Pneumonia After Hepatectomy

**DOI:** 10.1007/s11605-015-2869-1

**Published:** 2015-06-24

**Authors:** Kazuhiko Sakamoto, Takao Tamesa, Yoshihiro Tokuhisa, Satoshi Matsukuma, Yukio Tokumitsu, Yoshinari Maeda, Shigeru Takeda, Tomio Ueno, Shigeru Yamamoto, Shigefumi Yoshino, Shoichi Hazama, Hiroaki Nagano, Masaaki Oka

**Affiliations:** Department of Digestive Surgery and Surgical Oncology, Yamaguchi University Graduate School of Medicine, 1-1-1, Minami-Kogushi, Ube, Yamaguchi 755-8505 Japan

**Keywords:** Postoperative pneumonia, Hepatectomy

## Abstract

**Background:**

The purpose of this study was to retrospectively evaluate microbial examination of sputum on postoperative day one (POD1) and to determine risk factors for postoperative pneumonia (POP) after hepatectomy.

**Methods:**

Two hundred ninety-four patients who expectorated sputum on POD1 after hepatectomy between 2003 and 2014 were investigated. Sputum samples were submitted for microbial examination. Risk factors for POP were identified using multivariable analysis.

**Results:**

One hundred fifty-eight (53.7 %) of 294 patients had bacteria in their sputum on POD1. POP was observed in 24 (8.2 %) patients, with increased mortality in the patients with POP (0.74 vs 12.5 %, *p* < 0.01). Multivariate analysis demonstrated that a Brinkman index of >400 and bacteria in sputum on POD1 were independent risk factors for POP. Bacterial homology in sputum obtained on POD1 and onset day of POP was found in 13 of the 24 (54.2 %) patients with POP. In particular, in 13 patients with POP caused by methicillin-resistant *Staphylococcus aureus* or *Pseudomonas aeruginosa*, homology was confirmed in 9 patients (69.2 %).

**Conclusion:**

A Brinkman index ≥400 and bacteria in sputum on POD1 increased the risk of POP. Presence of bacteria in sputum on POD1 may be useful in determining early treatment against POP after hepatectomy.

## Introduction

Postoperative complications, such as pneumonia, aspiration, and acute respiratory distress syndrome, are usually related to the preoperative status of the patient. These postoperative pulmonary complications after surgery are associated with morbidity and have been shown to increase the hospital length-of-stay.^1^,^2^ The reported incidence of postoperative pulmonary complications varies between 10 and 80 %.^3^ With respect to postoperative pneumonia (POP) after hepatectomy, the rate has been reported as between 9.2 and 13 %.^4^–^7^ Perioperative strategies have been introduced for the purpose of reducing postoperative pulmonary complications.^8^,^9^ Carrel et al. reported that early pneumonia after coronary artery bypass grafting (CABG) is most probably caused by microorganisms that colonize the respiratory tract before operation, and the risk of early pneumonia after CABG is higher in patients with positive preoperative tracheal aspirates than in those with negative preoperative tracheal aspirates.^10^ With reference to this report by Carrel et al., we have been performing microbial examination of sputum on postoperative day (POD) 1.

The aims of the present study were to assess the incidence of bacteria in sputum on POD1, assess the predictive value of bacteria-positive sputum on POD1 for the occurrence of POP after hepatectomy, clarify whether bacteria-positive sputum on POD1 correlates with the results of bacteriology analysis performed after the onset of POP, and evaluate risk factors for the development of POP after hepatectomy.

## Methods

### Patients

We conducted a retrospective analysis of 363 patients undergoing hepatectomy without laparoscopy or simultaneous procedures such as biliary reconstruction, gastrointestinal resection, or splenectomy between 2003 and 2014 in the Department of Digestive Surgery and Surgical Oncology, Yamaguchi University Graduate School of Medicine. Sixty-nine patients who were not able to expectorate sputum on POD1 were excluded; therefore, 294 patients who expectorated sputum on POD1 were investigated. Written informed consent was obtained from all study patients.

### Preoperative Respiratory Management and Assessment

At the first visit to our hospital, all patients were instructed to quit smoking, and respiratory rehabilitation was started at the same time. History of smoking, cigarette consumption, and Brinkman index were noted. Brinkman index was defined by the number of cigarettes smoked/day multiplied by the number of years.^11^ Respiratory function including vital capacity (VC) and forced expiratory volume in 1.0 s (FEV1.0) were measured by spirometry preoperatively.

### Surgical Procedures

Intermittent pedicle clamping (Pringle maneuver) or selective clamping of the pedicles for the segment to be resected was performed only in cases of significant bleeding. Parenchymal transection was performed using cautery with irrigation forceps (CIF) between 2003 and 2006.^12^ We introduced an electrosurgical device (VIO 300D; ERBE Elektromedizin, Tübingen, Germany) containing the monopolar soft-coagulation and bipolar clamp coagulation systems beginning in 2007.^13^,^14^

### Postoperative Management

As prophylactic antibiotics, the patients received first-generation cephem antibiotics by intravenous infusion for 30 min before the operation. An additional dose was administered if the operation was prolonged beyond 3 h. Additionally, the patients continued to receive the same antibiotics plus further treatment at 12-h intervals for a total of 1–3 days. After induction of anesthesia, a nasogastric tube was placed until the morning of POD1 in all patients.

Sputum samples were submitted for microbial examination (Gram stain and semiquantitative bacteriologic cultures) on POD1. Chest X-rays were performed on POD1 and POD4 routinely. For the patients suspected to have postoperative pneumonia, chest X-rays and computed tomography (CT) scans were obtained, and sputum specimens were submitted immediately for microbial examination at that time.

### Definition of Postoperative Pneumonia

POP was diagnosed if purulent sputum was collected and yielded growth of relevant pathogens on culture, and if at least two of the following additional criteria were met: (1) white blood cell count >10,000 /mm,^3^ (2) temperature >38 °C, and (3) new or increasing lung infiltrate on conventional chest X-ray.

### Statistical Analysis

Hepatic fibrosis staging was classified by the pathologists as follows: no fibrosis (F0), portal fibrosis without septa (F1), portal fibrosis with few septa (F2), numerous septa without cirrhosis (F3), and cirrhosis (F4).^15^ To identify the perioperative risk factors for POP, the following variables were included: 9 patient variables (age, sex, body mass index (BMI), fibrosis staging, glucose intolerance, indocyanine green retention rate at 15 min (ICG-R15), VC, FEV, and sputum on POD1) and 3 surgical variables (type of hepatectomy, operation time, and blood loss). Continuous data were expressed as median and range values and were analyzed using the Mann-Whitney *U* test. Categorical data were analyzed using the *χ*^2^ test. Variables with *p* < 0.05 in the univariate analysis that were potentially predictive of POP were then entered into the multivariate logistic regression model. The cut-off value of continuous variables was determined using receiver operating characteristic (ROC) curves, and the optimal cut-off points were determined using the minimum distance from the upper-left corner to any point on the ROC curve. The odds ratio (OR) and 95 % confidence interval (CI) were also calculated. A value of *p* < 0.05 was considered statistically significant. All statistical calculations were performed with the IBM SPSS Statistics version 22.0 software package (IBM Japan Inc., Tokyo, Japan).

## Results

Among the 294 study patients, the median age was 69 years (range, 32–85 years) and the proportion of male patients was 78.2 % (*n* = 230). The majority of patients had hepatocellular carcinoma (*n* = 231: 78.6 %). Metastatic liver tumor was present in 38 (12.9 %) patients. A smoking history was confirmed in 198 (67.3 %) patients, and the median Brinkman index was 405 (range, 0–2200). The median VC and FEV1.0 were 3.33 L (range, 1.28–6.14 L) and 2.47 L (range, 0.72–4.82 L), respectively.

One hundred fifty-eight (53.7 %) of 294 patients had bacteria present in their sputum on POD1 (Table 1). *Haemophilus* species in Gram-negative bacteria, *Staphylococcus* species in Gram-positive bacteria, and *Candida albicans* among fungi were the most commonly isolated organisms. *Pseudomonas aeruginosa* and methicillin-resistant *Staphylococcus aureus* (MRSA) were confirmed in 19 and 13 patients, respectively.

**Table 1:** Result of microbial examination of sputum on postoperative day 1 (*n* = 158)^a^

Gram-negative bacteria		Gram-positive bacteria		Fungi	
*Haemophilus spp*	33	*Staphylococcus spp*	52	*Candida albicans*	36
*Pseudomonas aeruginosa*	19	(MSSA)	(38)	*Candida glabrate*	18
*Enterobacter spp*	16	(MRSA)	(13)	*Candida tropicalis*	6
*Klebsiella spp*	12	*Streptococcus constellatus*	19	*Penicillium spp*	1
*Acinetobacter spp*	7	*Enterococcus spp*	8		
*Serratia marcescens*	4				
*Branhamella catarrhalis*	2				
*Escherichia coli*	2				
*Neisseria subflava*	2				
*Stenotrophomonas maltophilia*	2				
*Bordetella bronchiseptica*	1				
*Capnocytophaga spp*	1				
*Eikenella corrodens*	1				
*Kingella denitrificans*	1				
*Kluyvera ascorbata*	1				
*Morganella morganii*	1				
*Proteus vulgaris*	1				

*MSSA* methicillin-sensitive *Staphylococcus aureus*, *MRSA* methicillin-resistant *Staphylococcus aureus*

^a^There are duplicated cases

POP was observed after hepatectomy in 24 (8.2 %) patients (Table 2). POP was predominantly caused by *P. aeruginosa* (5 patients) among Gram-negative bacteria and MRSA (8 patients) among Gram-positive bacteria, respectively. Bacterial homology in sputum obtained on POD1, and the day of onset of POP was noted in 13 (54.2 %) of the 24 patients with POP. In particular, there were 13 patients with POP caused by *P. aeruginosa* or MRSA, of whom homology was confirmed in 9 of those patients (69.2 %). The presence of bacteria in sputum on POD1 showed 91.7 % sensitivity, 49.6 % specificity, 13.9 % positive predictive value, and 98.5 % negative predictive value for prediction of POP. POP developed in only 2 (1.5 %) of 136 patients who expectorated sputum without bacteria, and in only 1 (1.4 %) patient among 69 patients who did not expectorate sputum on POD1.

**Table 2:** Characteristics of postoperative pneumonia

	Postoperative pneumonia (*n* = 24)	
Median duration from operation to diagnosis of POP (day)	5 (1–44)	
Microbial examination of sputum on onset day of pneumonia^a^		
Gram-negative bacteria	*Pseudomonas aeruginosa* *Enterobacter spp* *Acinetobacter spp* *Klebsiella spp* *Citrobacter freundii* *Proteus mirabilis* *Serratia marcescens*	5 3 2 2 1 1 1
Gram-positive bacteria	*Staphylococcus spp* (MRSA) (MRSE) *Enterococcus spp* *Streptococcus spp*	10 (8) (1) 2 2
Fungi	*Candida albicans* unknown	1 2
Bacterial homology in sputum obtained on POD1 and onset day of pneumonia	13 (54.2 %)	

Data are presented as absolute numbers or median (range, minimum-maximum)

*POP* postoperative pneumonia, *MRSA* methicillin-resistant *Staphylococcus aureus*, *MRSE* methicillin-resistant *Staphylococcus epidermidis*, *POD* postoperative day

^a^There are duplicated cases

Comparison of perioperative factors between groups with and without POP revealed a significantly increased risk of POP in patients in association with serum albumin (*p* = 0.02), Brinkman index (*p* = 0.02), VC (*p* = 0.042), FEV1.0 (*p* < 0.01), and sputum with bacteria on POD1 (*p* < 0.01), respectively. POP significantly increased mortality (0.74 vs 12.5 %, *p* < 0.01) (Table 3). ROC curve analysis indicated that the optimal cut-offs for serum albumin, the Brinkman index, VC, and FEV1.0 were 4.0 g/dL (79.2 % sensitivity and 47.8 % specificity), 400 (79.2 % sensitivity and 48.9 % specificity), 3.18 L (66.7 % sensitivity and 60.4 % specificity), and 2.44 L (75.0 % sensitivity and 54.1 % specificity), respectively, for the occurrence of POP. Multivariate analysis demonstrated that Brinkman index ≥400 and bacteria in sputum on POD1 were independent predictors of POP (Table 4).

**Table 3:** Patient characteristics with and without postoperative pneumonia

	Pneumonia (−) (*n* = 270)		Pneumonia (+) (*n* = 24)		*p* value
Age	69	69 (32–85)	71	(57–80)	0.29
Gender					
Male	211	(91.7 %)	19	(8.3 %)	0.91
Female	59	(92.2 %)	5	(7.8 %)	
Fibrosis staging					
F 0-1	98	(94.2 %)	6	(5.8 %)	0.27
F 2-4	172	(90.5 %)	18	(9.5 %)	
Glucose intolerance					
No	179	(92.7 %)	14	(7.3 %)	0.43
Yes	91	(90.1 %)	10	(9.9 %)	
ICG-R15 (%)	13.9	(2.0–52.6)	14.7	(3.6–48.1)	0.27
Serum albumin (g/dl)	3.9	(2.1–5.1)	3.7	(2.8–4.6)	0.02
Total bilirubin level (mg/dl)	0.8	(0.1–2.2)	0.7	(0.3–1.7)	0.16
Prothrombin activity (%)	88.2	(61.3–150)	84.7	(53.8–121.5)	0.27
Brinkman index	400	(0–2200)	800	(0–1600)	0.02
VC (L)	3.34	(1.28–6.12)	2.97	(2.12–4.34)	0.042
FEV1.0 (L)	2.51	(0.99–4.82)	2.22	(0.72–2.93)	<0.01
Sputum on POD1					
Without bacteria	134	(98.5 %)	2	(1.5 %)	<0.01
With bacteria	136	(86.1 %)	22	(13.9 %)	
Type of hepatectomy					
(Extended) hemihepatectomy or sectionectomy	133	(92.4 %)	11	(7.6 %)	0.75
Segmentectomy or partial hepatectomy	137	(91.3 %)	13	(8.7 %)	
Operation time (min)	358	(76–1028)	328	(155–560)	0.7
Blood loss (g)	470	(13–9425)	735	(35–2050)	0.12
Mortality					
No	268	(92.7 %)	21	(7.3 %)	<0.01
Yes	2	(40.0 %)	3	(60.0 %)	

Data are presented as absolute numbers (percentage) or median (range, minimum-maximum)

*HCC* hepatocellular carcinoma, *ICG-R15* indocyanine green retention rate at 15 min, *VC* vital capacity, *FEV1.0* forced expiratory volume in 1.0 s

**Table 4:** Multivariate logistic regression predicting development of postoperative pneumonia

Factor	OR	95 % CI	*p* value
Serum albumin (g/dl) (<3.9 vs. ≥4.0)	2.88	0.98–8.52	0.06
Brinkman index (≥400 vs. <400)	4.29	1.44–12.8	<0.01
VC (<3.18 vs. ≥3.18)	1.88	0.55–6.36	0.31
FEV1.0 (<2.44 vs. ≥2.44)	2.87	0.79–10.4	0.11
Bacteria in sputum on POD1 (yes vs. no)	9.43	2.11–42.0	<0.01

*OR* odds ratios, *CI* confidence interval, *VC* vital capacity, *FEV1.0* forced expiratory volume in 1.0 s, *POD* postoperative day

## Discussion

In the current study, the incidence of POP after hepatectomy was 8.2 %. In addition, patients with POP had significantly increased mortality. Although the homology between species bacteria in sputum on POD1 and caused pneumonia was not high (54 %), sensitivity (91.7 %) and negative predictive value (98.5 %) of the presence of bacteria in sputum on POD1 to predict POP were high. According to multivariate analysis, two independent risk factors for POP after hepatectomy were statistically significant: Brinkman index ≥400 and bacteria in sputum on POD1. Although several risk factors have already been reported for the occurrence of pulmonary complications,^4^,^6^,^16^,^17^ the risk factors have varied between studies due to differences in the study objectives, the definition used for pulmonary complications, and the type of surgery.

Previous studies have shown that the risk of postoperative pulmonary complications is mildly elevated among smokers.^18^,^19^ In the current study, 67.3 % of the patients had a history of smoking, and about half of them had a Brinkman index ≥400. Kojima et al. reported that a Brinkman index ≥400 relates to a risk for decreased respiratory function.^20^ In the field of esophageal surgery, smoking has been reported to be a risk factor for pulmonary complications, and it has also been reported that tobacco cessation and preoperative respiratory rehabilitation are expected to reduce the occurrence of complications. Two randomized trials have studied the impact of perioperative smoking intervention programs.^21^,^22^ Because both trials studied patients undergoing low-risk procedures, the trials were insufficiently powered to show a difference in pulmonary complication rates. A previous cohort study showed paradoxically higher postoperative pulmonary complication rates for smokers who stopped or reduced smoking within 2 months before noncardiothoracic surgery.^23^ Preoperative cigarette cessation to reduce the occurrence of pulmonary complications has been controversial.

Carrel et al. reported the relationship between preoperative tracheal aspirates of patients undergoing CABG and POP.^10^ Of 500 patients, 91 (18.2 %) had a positive Gram stain, and the incidence of POP was significantly higher in patients with preoperative positive tracheal aspirates (15.3 %) than in patients with negative tracheal aspirates (3.6 %; *p* < 0.01). In patients experiencing POP, there was a high correlation between the bacteria in preoperative aspirates and the bacteria observed when POP developed. A positive tracheal aspirate was one of the significant risk factors for developing postoperative pneumonia. In comparing the present study and the report of Carrel et al., although there is no difference in the incidence of POP in the patients who had bacteria in their sputum on POD1 (22/158: 13.9 %), the incidence of bacteria-positive sputum on POD1 (158/294: 53.7 %) was higher in the present study than in the Carrel report. The sputum specimens on POD1 may have been affected by surgical stress or intraoperative antibiotic prophylaxis. Conversely, we believe there is significance to the sample collection after the bacterial flora has been changed by these modifications. When the presence of bacteria in sputum on POD1 was confirmed and POP subsequently developed, this information allowed for the selection of an appropriate therapeutic antimicrobial drug against POP.

In conclusion, the current study examined the frequency of POP after hepatectomy and investigated the risk factors for POP. The patients with a Brinkman index of ≥400 and bacteria in sputum on POD1 require careful pulmonary care and should be closely monitored during the perioperative period. The information obtained from the patient’s sputum on POD1 may be useful in the treatment of POP. We did not have any protocol to prevent POP against carrier having bacteria in sputum on POD1 in this study. The protocol that prophylactic antibiotics for POP is administered using the information of bacteria in sputum on POD1 may be considered prospectively.
